# Benign Evolution of Complex Regional Pain Syndrome (CRPS) Type 1 in Patients Treated with Intravenous Neridronate: A Single-Center Real-Life Experience

**DOI:** 10.3390/ph17111500

**Published:** 2024-11-08

**Authors:** Jacopo Ciaffi, Gianluca Festuccia, Claudio Ripamonti, Luana Mancarella, Veronica Brusi, Federica Pignatti, Lucia Lisi, Lisa Berti, Piero Ruscitti, Cesare Faldini, Francesco Ursini

**Affiliations:** 1 Medicine & Rheumatology Unit, IRCCS Istituto Ortopedico Rizzoli, 40136 Bologna, Italy; claudio.ripamonti@ior.it (C.R.); luana.mancarella@ior.it (L.M.); veronica.brusi@ior.it (V.B.); federica.pignatti@ior.it (F.P.); lucia.lisi@ior.it (L.L.); francesco.ursini2@unibo.it (F.U.); 2 Department of Biomedical and Neuromotor Sciences (DIBINEM), University of Bologna, 40127 Bologna, Italy; lisa.berti@ior.it (L.B.); cesare.faldini@ior.it (C.F.); 3 Physical Medicine and Rehabilitation Unit, IRCCS Istituto Ortopedico Rizzoli, 40136 Bologna, Italy; gianluca.festuccia@ior.it; 4 Department of Biotechnological and Applied Clinical Sciences, University of L’Aquila, 67100 L’Aquila, Italy; piero.ruscitti@univaq.it; 5 1st Orthopaedic and Traumatologic Clinic, IRCCS Istituto Ortopedico Rizzoli, 40136 Bologna, Italy

**Keywords:** chronic regional pain syndrome, neridronate, real-life, PROMIS-29

## Abstract

Objective: To investigate the long-term effects of intravenous neridronate treatment in patients with complex regional pain syndrome type 1 (CRPS) in a real-life setting. Methods: We conducted a retrospective study on consecutive CRPS patients treated at our hospital from February 2018 to July 2023. All were treated within three months of the onset of CRPS symptoms. The Patient-Reported Outcomes Measurement Information System 29-Item Health Profile (PROMIS-29) version 2.1 was administered. The main outcome of interest was the evolution of the PROMIS-29 scores from baseline to the last follow-up visit. Patients were categorized as “complete responders” or “non-complete responders”. The association of clinical and demographic variables with a complete response was analyzed using chi-square tests and univariate logistic regression. Results: Thirty-six patients were included, with a median follow-up time of 4.8 years. A significant improvement was noted in the mean numerical pain rating scale (from 6.4 ± 1.9 to 3.1 ± 2.4, *p* < 0.001), as well as across all PROMIS-29 domains. Physical function improved from 34.2 ± 4.9 to 49.2 ± 9.9, *p* < 0.001; anxiety from 58.0 ± 6.7 to 49.6 ± 6.9, *p* < 0.001; depression from 55.3 ± 6.3 to 47.7 ± 6.6, *p* < 0.001; fatigue from 55.7 ± 7.7 to 50.9 ± 8.7, *p* < 0.001; sleep disturbance from 53.8 ± 6.8 to 51.3 ± 6.6, *p* = 0.034; social roles and activities from 41.8 ± 5.2 to 51.8 ± 8.9, *p* < 0.001; and pain interference from 64.1 ± 5.9 to 52.4 ± 9.9, *p* < 0.001. The likelihood of achieving a complete response was associated with the male sex, foot or ankle injuries (compared to hand and wrist injuries), and a younger age. No association was found with the type of inciting event or with the body mass index. Conclusions: Our real-life data indicate that early treatment with neridronate leads to substantial benefits in patients affected by CRPS type 1. The strongest responses are seen in young patients, males, and those with lower limb involvement.

## 1. Introduction

Complex regional pain syndrome (CRPS) is a condition characterized by pain, swelling, vasomotor abnormalities, and sensory disturbances that are disproportionate to the inciting event [1]. The disease can be categorized into two subtypes [2]. CRPS type 1 (CRPS-1), previously named “reflex sympathetic dystrophy”, usually occurs after trauma or surgery to the upper or lower limbs without any nerve injury, while CRPS type 2 (CRPS-2), also known as “causalgia”, develops in patients with identifiable peripheral nerve damage [2].

Bisphosphonates play an established role in treating CRPS-1, with evidence of their efficacy primarily coming from seven randomized controlled trials conducted over the last three decades [3,4,5,6,7,8,9,10]. Nitrogen-containing bisphosphonates, such as alendronate, neridronate, and pamidronate, primarily inhibit farnesyl pyrophosphate synthase, a key enzyme of the mevalonate pathway, thereby inactivating osteoclast activity and potentially inducing osteoclast apoptosis [11,12].

The use of parenteral bisphosphonates as first-line agents for the treatment of CRPS-1 is well supported by the literature, although the exact mechanisms of their efficacy remain poorly understood [13]. There is no definitive evidence that CRPS-1 is characterized by enhanced osteoclastic activity, suggesting that the clinical improvement observed in CRPS-1 patients treated with bisphosphonates may not be entirely related to the antiresorptive properties of these agents [14,15].

In particular, studies on neridronate, a bisphosphonate structurally similar to alendronate and pamidronate, have shown the most convincing results, mainly in patients with early disease [8,11]. Two randomized controlled trials demonstrated the efficacy of both the intravenous infusion and intramuscular administration protocols of neridronate in patients with CRPS-1, with no emerging safety concerns [8,9].

Based on these positive results, neridronate has been registered in Italy since 2015 for the treatment of CRPS-1. Since then, the intravenous treatment schedule of 100 mg every third day for four consecutive times has been widely used in Italy, although long-term results have seldom been reported [16,17]. Varenna et al. showed that patients treated with either intravenous or intramuscular neridronate experienced sustained improvements in pain and functional measures over a follow-up period of 12 months [16].

Longer-term effectiveness results were presented by Adami et al., who reported data on 78 patients followed for a median of 3.6 years, confirming that the rapid benefits conferred by intravenous neridronate treatment can be maintained in a substantial proportion of patients [17]. Additionally, the authors described that the type of predisposing event, affected site, gender, and early response to treatment can be used as predictors of the long-term response [17]. No other study has explored the long-term outcomes of intravenous neridronate in a real-life setting in terms of both effectiveness and safety. Thus, we aimed to investigate the long-term effects of neridronate treatment in a real-life setting, assessing changes in pain intensity and various domains of quality of life.

## 2. Results

### 2.1. Patients’ Characteristics

Of the 47 contacted patients, 11 either did not respond (*n* = 4) or were not available (*n* = 7) for an in-person visit. Ultimately, 36 patients (23 women and 13 men) were included in the study (Table 1). The median time between neridronate treatment and the last follow-up data collection was 4.8 years (IQR 3.1–5.4 years). The mean age was 62.7 ± 9.4 years, and the mean time from the onset of CRPS symptoms to the first neridronate infusion was 9.4 ± 2.3 weeks.

Eight patients experienced an acute-phase reaction after neridronate infusion, and, in one case, the treatment cycle could not be completed. Therefore, all but one patient received a total dose of 400 mg of neridronate. All patients received calcium and vitamin D supplementation starting two weeks before the first infusion. Calcium was continued for two weeks after the infusion cycle, while vitamin D supplementation was maintained for at least three months in all cases. In most patients, the lower limb was the injured site, with foot and/or ankle involvement observed in 28 cases (78%). The “warm” disease subtype (*n* = 34, 94%) was more common than the “cold” subtype (*n* = 2, 6%).

The inciting events were either fractures (*n* = 10; 28%), surgery (*n* = 7; 19%), fractures and surgery (*n* = 3; 8%), trauma without a fracture (*n* = 6; 17%), or mechanical overload (*n* = 2; 6%), while no predisposing event could be identified in eight cases (22%).

### 2.2. Evolution of Patient-Reported Outcomes Measurement Information System 29-Item Health Profile (PROMIS-29)

Each PROMIS-29 domain was analyzed separately. The mean numeric pain rating scale (NPRS) score was 6.4 ± 1.9 before treatment with neridronate and improved to 3.1 ± 2.4 at the last follow-up visit (*p* < 0.001). The mean physical function score was 34.2 ± 4.9 before treatment and improved to 49.2 ± 9.9 at the last follow-up visit (*p* < 0.001). The mean anxiety score was 58.0 ± 6.7 before treatment and improved to 49.6 ± 6.9 at the last follow-up visit (*p* < 0.001). The mean depression score was 55.3 ± 6.3 before treatment and improved to 47.7 ± 6.6 at the last follow-up visit (*p* < 0.001). The mean fatigue score was 55.7 ± 7.7 before treatment and improved to 50.9 ± 8.7 at the last follow-up visit (*p* < 0.001). The mean sleep disturbance score was 53.8 ± 6.8 before treatment and improved to 51.3 ± 6.6 at the last follow-up visit (*p* = 0.034). The mean score for the ability to participate in social roles and activities was 41.8 ± 5.2 before treatment and improved to 51.8 ± 8.9 at the last follow-up visit (*p* < 0.001). The mean pain interference score was 64.1 ± 5.9 before treatment and improved to 52.4 ± 9.9 at the last follow-up visit (*p* < 0.001).

**Figure 1:**
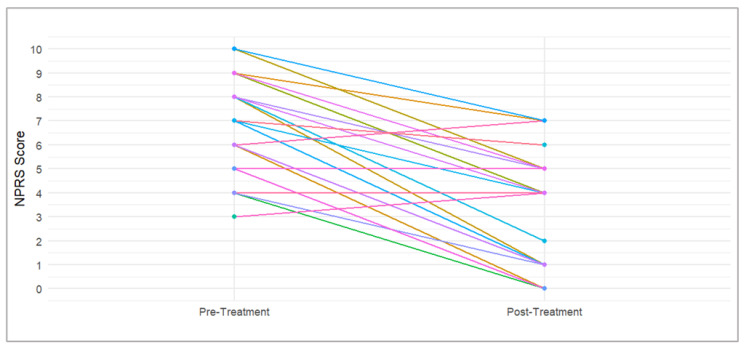
Spaghetti plot showing individual patient trajectories of NPRS improvement.

**Figure 2:**
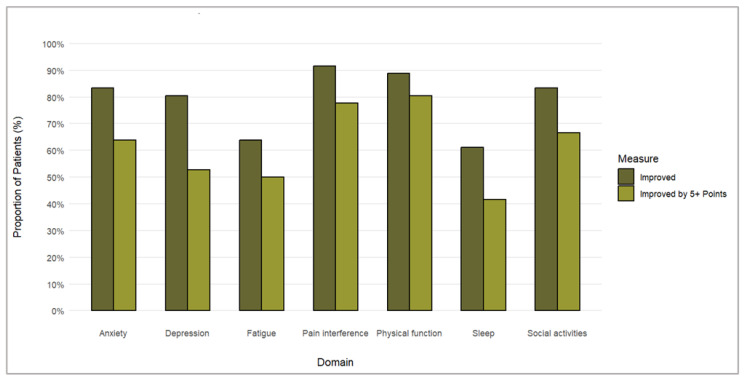
Proportions of patients achieving improvements and clinically meaningful improvements in each PROMIS-19 domain.

The NPRS improved in 32 patients (89%), with the improvement exceeding the minimal clinically important difference (MCID) in 25 cases (69%). The patterns of NPRS improvement in individual patients are shown in Figure 1. Analyzing the proportion of patients with improvements and clinically meaningful improvements in each PROMIS-29 component (Figure 2), we found the following.

- Physical function: 89% improvement, 81% clinically meaningful improvement;
- Anxiety: 83% improvement, 64% clinically meaningful improvement;
- Depression: 81% improvement, 53% clinically meaningful improvement;
- Fatigue: 64% improvement, 50% clinically meaningful improvement;
- Sleep disturbances: 61% improvement, 42% clinically meaningful improvement;
- Ability to participate in social roles and activities: 83% improvement, 67% clinically meaningful improvement;
- Pain interference: 92% improvement, 78% clinically meaningful improvement.

**Figure 3:**
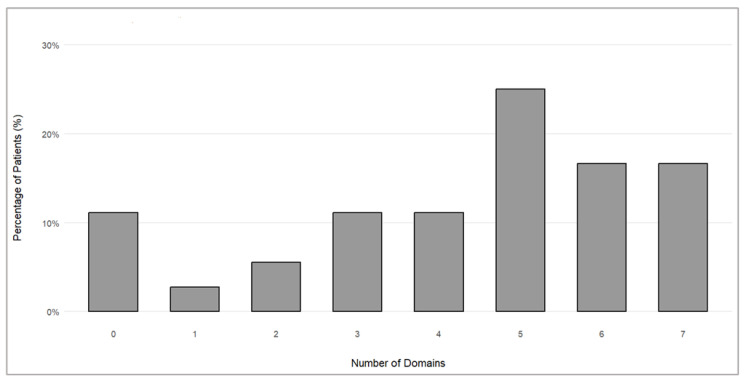
Proportions of patients achieving clinically meaningful improvements in different numbers of PROMIS-29 domains.

Most patients achieved the MCID across multiple PROMIS-29 scales. Specifically, a meaningful improvement was seen in five domains for nine patients, six domains for six patients, and seven domains for six patients (Figure 3). In three cases, there was no improvement in either the NPRS or any of the PROMIS-29 domains.

### 2.3. Characteristics of PROMIS-29 Complete Responders

Overall, 56% of the patients were complete responders according to the predefined PROMIS-29 criteria. A larger proportion of patients achieving a complete response was observed in males compared to females (85% vs. 39%, *p* = 0.008) and in patients with foot or ankle injuries compared to those with hand and wrist injuries (64% vs. 25%, *p* = 0.048). Additionally, there was a significant negative association between increasing age and the response to treatment, with an odds ratio (OR) of 0.85 (95% CI 0.76 to 0.94, *p* = 0.002). An opposite trend towards significance was observed for the body mass index (BMI), with an OR of 1.30 (95% CI 0.98 to 1.72, *p* = 0.070).

When comparing CRPS-1 cases triggered by fractures and/or surgery to those caused by trauma, overload, or no predisposing events, no significant differences were found in the likelihood of being complete responders at follow-up (55% vs. 56%, *p* = 0.940).

### 2.4. Follow-Up Pain Catastrophizing Scale (PCS), Short-Form McGill Pain Questionnaire-2 (SF-MPQ-2), Pain Self-Efficacy Questionnaire (PSEQ), and EuroQoL-5 Dimensions (EQ-5D)

Data for the PCS, PSEQ, SF-MPQ-2, and EQ-5D were not collected at baseline. At the last follow-up visit, the mean PCS score was 17.3 ± 9.2, the mean PSEQ score was 44.6 ± 14.1, and the mean SF-MPQ-2 score was 1.4 ± 1.5. Regarding the EQ-5D, the mean utility score was 0.70 ± 0.31, and the mean visual analog scale (VAS) score was 71.9 ± 19.1.

Strong associations were observed between the follow-up scores, with higher Pearson’s correlation coefficients (r) seen between the EQ-5D VAS and EQ-5D utility score (r = 0.79, *p* < 0.001), EQ-5D VAS and PSEQ (r = 0.75, *p* < 0.001), EQ-5D VAS and NPRS (r = −0.70, *p* < 0.001), NPRS and SF-MPQ-2 (r = 0.67, *p* < 0.001), EQ-5D utility score and PSEQ (r = 0.63, *p* < 0.001), and NPRS and PSEQ (r = −0.63, *p* < 0.001).

Comparing the results of the PCS, PSEQ, SF-MPQ-2, and EQ-5D between patients categorized as “complete responders” and those who were not according to the pre-defined PROMIS-19 MCID criteria, significant differences were noted. Complete responders had higher EQ-5D utility scores (0.83 ± 0.18 vs. 0.54 ± 0.37, *p* = 0.004), higher PSEQ scores (50.0 ± 10.1 vs. 37.8 ± 15.8, *p* = 0.012), and lower SF-MPQ-2 total values (0.78 ± 0.78 vs. 2.28 ± 1.76, *p* <0.001). Conversely, no difference was observed in the PCS (15.0 ± 8.7 vs. 19.4 ± 9.6, *p* = 0.856) and EQ-5D VAS (81.2 ± 13.4 vs. 60.3 ± 19.0, *p* = 0.160) between complete responders and non-complete responders.

## 3. Discussion

Although neridronate has been approved in Italy for the treatment of CRPS for a decade, data on its long-term effectiveness remain limited [16,17]. Only two articles have reported sustained benefits up to 3.6 years post-infusion [16,17]. To expand on these findings, we conducted a retrospective study on 36 CRPS-1 patients, followed at our academic hospital for a median time period of 4.8 years.

Using PROMIS-29 as the main outcome of interest and defining the response according to the NPRS and PROMIS-29 scales, we found an overall benign course of CRPS after neridronate treatment, with 56% of patients achieving a complete response. Neridronate infusions were generally well tolerated, with the typical acute-phase reaction, characterized by diffuse musculoskeletal pain and fever, observed in eight patients and managed with paracetamol. Only one patient was unable to complete the treatment cycle.

In line with prior research, we observed that male patients and those with lower extremity involvement had better outcomes [17]. Furthermore, a significant association was found between age and the treatment response, with older patients being less likely to achieve a meaningful improvement. Contrasting results have been reported in the literature regarding the role of inciting events. In a retrospective chart review of 194 patients treated with different bisphosphonates (neridronate, pamidronate, or clodronate), Varenna et al. found a greater percentage of responders among those with a history of fractures compared to patients whose predisposing event was either trauma without a fracture, surgery, or other events [18]. Adami et al. reported the absence of an inciting event as a predisposing factor for an excellent response to neridronate treatment, while the authors did not find significant differences between patients whose CRPS was triggered by a fracture compared to those triggered by surgery or trauma without a fracture [17].

Although our study’s limited sample size hindered detailed sub-analyses, we found no significant difference in the response rates when categorizing patients into major (fracture/surgery) versus minor (trauma without fracture/mechanical overload/none) inciting events. Larger studies are needed to further investigate the factors influencing the treatment outcomes.

Another limitation is that only PROMIS-29 data were collected at both baseline and follow-up. PROMIS-29 is extensively used in musculoskeletal research and has been in use at our institution for a long period of time [19,20,21]. The other tools suggested by the IASP were integrated into our clinical practice at the end of 2021, preventing the possibility of comparing changes from pre-treatment to post-treatment. However, the PROMIS-29 NPRS at the last follow-up was strongly correlated with the PSEQ, SF-MPQ-2, EQ-5D VAS, and EQ-5D utility scores. The post-treatment PCS scores indicated non-problematic thinking, despite no correlation with the NPRS. Overall, our results suggest that neridronate therapy improves multiple domains in CRPS patients, including pain, disease severity, social participation, self-efficacy, physical function, psychological distress, and catastrophizing.

However, 8% of patients did not experience any improvement in either pain intensity or any of the PROMIS-29 domains, highlighting the ongoing challenge of treating CRPS-1, even with early intervention. While we found that neridronate was effective in most cases, the results of our study suggest that individualized treatment approaches may be necessary for non-responders. Further research is needed to explore potential biomarkers or patient characteristics that could predict treatment success and guide personalized therapeutic strategies.

In addition to bisphosphonates, steroid therapy has been explored as a potential treatment option for CRPS type I, with several studies reporting positive outcomes. A recent randomized controlled trial demonstrated that corticosteroids significantly reduced pain and improved function in CRPS patients, likely due to their anti-inflammatory effects [22]. In particular, Kalita et al. have conducted multiple studies on the role of prednisolone in CRPS, showing its efficacy in reducing pain and improving motor function in post-stroke patients [22,23,24]. Their research also highlighted the importance of dose optimization, comparing different prednisolone dosages in CRPS management. Furthermore, a retrospective cohort study suggested that steroids may provide substantial benefits in the early stages of CRPS, although further research is needed to confirm their long-term efficacy [25]. While our study focused on bisphosphonates, it is important to acknowledge the potential of steroids as a complementary or alternative therapy, particularly for patients who may benefit from a multimodal treatment approach.

Notably, all patients in this study were treated within 3 months of CRPS symptom onset. The results may not be generalizable to all CRPS patients, particularly those with CRPS-2 or cases where treatment is delayed. The previous literature suggests that responders tend to have a shorter disease duration compared to non-responders [18]. Greater efficacy in the earliest phases of the disease has been hypothesized, when neridronate can interfere with local cytokine release and inhibit inflammatory processes [26,27]. Macrophages can be involved in achieving this effect [28]. Bisphosphonates target macrophages, impairing their differentiation from monocytes, affecting their proliferation and migration, and inducing apoptosis [29,30]. By interrupting the disease trajectory mediated by macrophages, neridronate may prevent the occurrence of the events leading to chronic CRPS, primarily represented by neurogenic inflammation, autonomic dysregulation, rapid bone turnover, and osteoporotic changes [31,32,33,34].

Disuse atrophy and bone demineralization are well-known consequences of CRPS, particularly in the affected limbs [35]. Kumar et al. highlighted the role of disuse atrophy in contributing to bone density loss in CRPS patients after stroke, further supporting the importance of considering bone health in CRPS management [36]. Although our study did not directly evaluate localized osteoporosis, this phenomenon is commonly observed in CRPS patients and may play a role in disease progression [37].

## 4. Materials and Methods

### 4.1. Study Design and Population

We conducted a retrospective study on consecutive patients fulfilling the diagnostic criteria for CRPS [38], who were treated with intravenous neridronate at the Rheumatology Unit of our Institution, IRCCS Istituto Ortopedico Rizzoli, Bologna, Italy, between February 2018 and July 2023. CRPS was categorized as “warm” when it presented with signs of inflammation, such as redness, warmth, and swelling in the affected area. In contrast, “cold” CRPS referred to cases where the affected area was cold, pale, and with reduced signs of inflammation [39]. Patients with evidence of major nerve damage suggestive of CRPS-2, and those with an onset of CRPS symptoms exceeding 3 months, were excluded. At our institution, CRPS patients receive in-person follow-ups at 6 and 12 months after completing intravenous neridronate treatment. Additional follow-up is performed when clinically necessary. All patients in this study were contacted by telephone to schedule an in-person follow-up visit between May 2024 and July 2024.

The research was conducted in compliance with the Declaration of Helsinki and its latest amendments [40]. The study protocol was approved by the local Ethics Committee (Comitato Etico Area Vasta Emilia Centrale, Bologna, Italy—CE AVEC: 327/2024/Oss/IOR, approval number 0009911). Written informed consent was obtained from all participants.

### 4.2. Outcome Measures

Starting from November 2016, at our institution, the PROMIS-29 version 2.1 has been administered in paper format to all patients with CRPS-1 [41]. The questionnaire is completed at baseline (i.e., before the first neridronate infusion) and at all subsequent follow-up visits.

Furthermore, in October 2021, the recommendations of the International Association for the Study of Pain (IASP) CRPS special interest group were incorporated into clinical practice [42]. As a result, the “Core Outcome Measures for Complex Regional Pain Syndrome Clinical Studies” (COMPACT) is proposed to all patients diagnosed with CRPS [42]. COMPACT uses various patient-reported outcomes, including PROMIS-29, to explore a broad range of domains, from pain and disease severity to physical function and catastrophizing [42]. The other COMPACT tools are the PCS, the SF-MPQ-2, the PSEQ, and the EQ-5D. All tools are available in the Italian language [43,44,45,46].

#### 4.2.1. Patient-Reported Outcomes Measurement Information System 29-Item Health Profile (PROMIS-29)

The main outcome of interest in our study was the PROMIS-29 version 2.1, a tool designed to evaluate 7 health status domains in patients suffering from chronic diseases [41]. The 7 domains are depression, anxiety, physical function, pain interference, fatigue, sleep disturbance, and the ability to participate in social roles and activities. The questionnaire consists of 4 items from each domain, with 5 response options per item. Additionally, there is a pain intensity item assessed through a 0–10 NPRS.

PROMIS-29 scores are evaluated using T-scores, where the reference population has a mean of 50 and a standard deviation of 10 [47]. The overall scales for each domain range from 20 to 80. Higher scores on the depression, anxiety, fatigue, sleep, or pain interference items represent worse symptoms, while higher scores on the physical function and social participation items are linked to improved function.

The MCID of PROMIS-29 in CRPS-1 has not been estimated, but, based on the available literature on chronic pain conditions, a change of 5 points in each scale can be considered as a reasonable MCID [48,49].

For the pain scale, a relative reduction of 50% and an absolute reduction of 3 cm on the VAS has been suggested as the MCID in CRPS-1 patients [50].

The license to use the questionnaire and the certified translation in Italian were obtained from the PROMIS Health Organization (Evanston, IL, USA).

#### 4.2.2. Pain Catastrophizing Scale

The PCS is a tool designed to evaluate the presence of catastrophic thoughts associated with pain in adults [51]. The extent of specific thoughts and feelings is rated on a scale from 0 (not at all) to 4 (all the time) across 13 items, with the overall score ranging from 0 to 52. Higher scores reflect greater pain catastrophizing. Scores below 30 suggest non-problematic thinking, while scores of 30 or above indicate problematic levels of catastrophic thinking. The PCS is recognized for its good validity, effectively measuring pain catastrophizing across diverse populations [51].

#### 4.2.3. Short-Form McGill Pain Questionnaire-2

The SF-MPQ-2 is an expanded and revised version of the Short-Form McGill Pain Questionnaire, consisting of 22 items that investigate 4 dimensions of neuropathic pain [52]. Each item is rated on a scale from 0 to 10, with higher scores representing more severe neuropathic pain. The total score ranges from 0 to 10 and is calculated as the mean of all items.

#### 4.2.4. Pain Self-Efficacy Questionnaire

The PSEQ is a questionnaire designed to measure a patient’s confidence in performing activities despite the presence of pain [53]. It consists of 10 items, each rated on a 0–6 scale. Total scores range from 0 to 60, with higher scores indicating greater pain self-efficacy.

#### 4.2.5. EuroQoL-5 Dimensions

The EQ-5D is a questionnaire consisting of two parts, used as a generic tool to measure health status [54]. In the first part, patients choose between three severity levels in 5 domains: mobility, self-care, usual activities, pain/discomfort, and anxiety/depression. The severity levels range from no problems to some problems and extreme problems. Preference-based, nation-specific weights are required to convert the response into a sum utility score [55]. For this study, we applied the Italian tariff, with results ranging from −0.39 to 1 [44]. A score of 1 suggests perfect health, whereas negative scores indicate a health status perceived as worse than death. The second part of the questionnaire is a 0–100 VAS where patients rate their health on the day from 0 (worst imaginable health) to 100 (best imaginable health).

### 4.3. Statistical Analysis

Descriptive statistics were used to report patients’ characteristics, utilizing the mean and standard deviation or median and interquartile range, as appropriate. Patients who experienced a reduction in the NPRS exceeding the MCID and at least 5 points of improvement in 5 or more domains of the PROMIS-29 were defined as “complete responders”. The chi-square test was employed to assess differences in categorical variables between patient groups, while univariate logistic regression was used to evaluate the associations between continuous variables and a complete response, providing ORs and 95% confidence intervals. The paired samples Student’s *t*-test was used to compare the difference in score in each PROMIS-29 domain and in NPRS from pre-treatment to the last follow-up visit. The two-sample Student’s *t* test was used to compare the PCS, PSEQ, SF-MPQ-2, and EQ-5D scores between complete responders and non-complete responders at the last follow-up visit. Pearson’s correlation coefficient (r) was calculated to estimate the correlations between continuous variables, specifically the different questionnaires scores at the last follow-up visit. A *p*-value < 0.05 was considered statistically significant.

The statistical analysis was performed using the R Statistical Software (version 4.4.0; R Foundation for Statistical Computing, Vienna, Austria) and the plots were created using the ‘ggplot2’ package [56].

## 5. Conclusions

In conclusion, although the primary mechanism of action of neridronate in treating CRPS remains controversial, our real-life data reinforce its effectiveness as a therapeutic option for this challenging condition. Early intervention with neridronate leads to significant and persistent benefits for a substantial proportion of patients, particularly younger individuals, males, and those with lower limb involvement. Our long-term results, with a median follow-up period of nearly 5 years, suggest that intravenous neridronate can provide curative effects in patients with acute CRPS, effectively preventing the progression to a chronic, disabling disease. These findings highlight the importance of early treatment and the need for further research to explore the factors influencing patient responsiveness and long-term outcomes.

## Figures and Tables

**Table 1:** Baseline characteristics of the study population, including demographic and clinical data.

Baseline Characteristics of the Study Population	*n* = 36
Sex, female, *n* (%)	23 (64)
Age (years), mean (SD)	63 (9)
BMI, mean (SD)	25 (3)
Never smokers, *n* (%)	21 (58)
Former smokers, *n* (%)	9 (25)
Current smokers, *n* (%)	6 (17)
CRPS duration (weeks), mean (SD)	9 (2)
Completed intravenous neridronate cycle, *n* (%)	35 (97)
Acute-phase reaction, *n* (%)	8 (22)
Type of CRPS-1	
Warm, *n* (%)	34 (94)
Cold, *n* (%)	2 (6)
Affected site	
Foot and ankle, *n* (%)	28 (78)
Hand and wrist, *n* (%)	8 (22)
Inciting event	
Fracture, *n* (%)	10 (28)
Surgery, *n* (%)	7 (19)
Fracture and surgery, *n* (%)	3 (8)
Trauma without fracture, *n* (%)	6 (17)
Mechanical overload, *n* (%)	2 (6)
None, *n* (%)	8 (22)
Presenting symptoms and signs	
Sensory, *n* (%)	27 (75)
Vasomotor, *n* (%)	10 (28)
Sudomotor/edema, *n* (%)	13 (36)
Motor/trophic, *n* (%)	31 (86)
Previous treatments	
PEMF, *n* (%)	13 (36)
Clodronate	9 (25)
Calcium and/or vitamin D supplements, *n* (%)	11 (31)
Physical therapy, *n* (%)	21 (58)
NSAIDs, *n* (%)	14 (39)
Orthoses, *n* (%)	12 (33)
None, *n* (%)	3 (8)
Comorbidities	
Autoimmune inflammatory rheumatic diseases, *n* (%)	4 (11)
Diabetes, *n* (%)	2 (6)
Hypertension, *n* (%)	11 (31)
Thyroid disease, *n* (%)	7 (19)
History of cancer, *n* (%)	3 (8)
Osteoporosis, *n* (%)	8 (22)

Legend: BMI: body mass index; CRPS: complex regional pain syndrome; NSAIDs: non-steroidal anti-inflammatory drugs; PEMF: pulsed electromagnetic field therapy; SD: standard deviation.

## Data Availability

The data presented in this study are available from the corresponding author upon reasonable request.
